# Anthropometric deficits and the associated risk of death by age and sex in children aged 6–59 months: A meta‐analysis

**DOI:** 10.1111/mcn.13431

**Published:** 2022-09-27

**Authors:** Susan Thurstans, Stephanie V. Wrottesley, Bridget Fenn, Tanya Khara, Paluku Bahwere, James A. Berkley, Robert E. Black, Erin Boyd, Michel Garenne, Sheila Isanaka, Natasha Lelijveld, Christine M. McDonald, Andrew Mertens, Martha Mwangome, Kieran S. O'Brien, Heather Stobaugh, Sunita Taneja, Keith P. West, Saul Guerrero, Marko Kerac, André Briend, Mark Myatt

**Affiliations:** ^1^ London School of Hygiene and Tropical Medicine London UK; ^2^ Emergency Nutrition Network Kidlington UK; ^3^ Epidemiology, Biostatistics and Clinical Research Centre, School of public Health Université libre de Bruxelles Brussels Belgium; ^4^ Centre for Tropical Medicine & Global Health University of Oxford Oxford UK; ^5^ Kenya Medical Research Institute (KEMRI) Centre for Geographic Medicine Research Coast (CGMRC) & KEMRI Wellcome Trust Research Programme Kilifi Kenya; ^6^ Department of International Health Johns Hopkins Bloomberg School of Public Health Baltimore USA; ^7^ USAID/Bureau of Humanitarian Assistance Washington DC USA; ^8^ Friedman School of Nutrition Science and Policy Tufts University Boston Massachusetts USA; ^9^ IRD UMI Résiliences Paris France; ^10^ Institut Pasteur Epidémiologie des Maladies Emergentes Paris France; ^11^ FERDI Université d'Auvergne Clermont‐Ferrand France; ^12^ MRC/Wits Rural Public Health and Health Transitions Research Unit, School of Public Health, Faculty of Health Sciences University of the Witwatersrand Johannesburg South Africa; ^13^ Harvard T. H. Chan School of Public Health Boston Massachusetts USA; ^14^ Epicentre Paris France; ^15^ Departments of Pediatrics, and Epidemiology & Biostatistics University of California San Francisco California USA; ^16^ Department of Nutrition University of California Davis USA; ^17^ Division of Epidemiology & Biostatistics University of California Berkeley USA; ^18^ Francis I. Proctor Foundation University of California San Francisco USA; ^19^ Action Against Hunger USA New York New York USA; ^20^ Center for Health Research and Development Society for Applied Studies New Delhi India; ^21^ United Nations Children's Fund (UNICEF) New York USA; ^22^ Center for Child Health Research, Faculty of Medicine and Medical Technology Tampere University Tampere Finland; ^23^ Department of Nutrition, Exercise, and Sports University of Copenhagen Fredericksberg Denmark; ^24^ Brixton Health, Llwyngwril Gwynedd Wales UK

**Keywords:** age, mortality, sex, stunting, underweight, wasting

## Abstract

Risk of death from undernutrition is thought to be higher in younger than in older children, but evidence is mixed. Research also demonstrates sex differences whereby boys have a higher prevalence of undernutrition than girls. This analysis described mortality risk associated with anthropometric deficits (wasting, underweight and stunting) in children 6–59 months by age and sex. We categorised children into younger (6–23 months) and older (24–59 months) age groups. Age and sex variations in near‐term (within 6 months) mortality risk, associated with individual anthropometric deficits were assessed in a secondary analysis of multi‐country cohort data. A random effects meta‐analysis was performed. Data from seven low‐or‐middle‐income‐countries collected between 1977 and 2013 were analysed. One thousand twenty deaths were recorded for children with anthropometric deficits. Pooled meta‐analysis estimates showed no differences by age in absolute mortality risk for wasting (RR 1.08, *p* = 0.826 for MUAC < 125 mm; RR 1.35, *p* = 0.272 for WHZ < −2). For underweight and stunting, absolute risk of death was higher in younger (RR 2.57, *p* < 0.001) compared with older children (RR 2.83, *p* < 0.001). For all deficits, there were no differences in mortality risk for girls compared with boys. There were no differences in the risk of mortality between younger and older wasted children, supporting continued inclusion of all children under‐five in wasting treatment programmes. The risk of mortality associated with underweight and stunting was higher among younger children, suggesting that prevention programmes might be justified in focusing on younger children where resources are limited. There were no sex differences by age in mortality risk for all deficits.

## Key points

- There is a high risk of mortality associated with child wasting. We found no difference in mortality risk between children 6–23 months and children 24–59 months, indicating the need to include all children under 5 years in wasting treatment programmes.
- For underweight and stunting, younger children had a significantly higher risk of mortality than older children. Where resources are limited, prevention programmes may be justified in targeting younger children.
- Despite sex differences in the prevalence of wasting, stunting and underweight, there were no differences in mortality risk between girls and boys in both younger and older age groups.

## 1 INTRODUCTION

Addressing all forms of undernutrition remains a public health priority for achieving the 2030 Sustainable Development Goals. Worldwide, 149 million children under 5 years of age are stunted (have a height‐for‐age z‐score < −2) and 45 million are wasted (have a weight‐for‐height z‐score < −2) (United Nations Children's Fund, World Health Organisation, The World Bank Group, 2021) with 15.9 million experiencing concurrent wasting and stunting (Global Nutrition Report, 2018). Evidence shows that, even in mild forms, anthropometric deficits are associated with increased mortality risk in children under five (Olofin et al., 2013).

The first 1000 days of life is a critical phase characterised by rapid growth and neurodevelopment, high nutrition requirements, increased susceptibility to infections, and full dependency on others to meet care, nutrition and social interaction requirements (Martorell, 2017). Younger children (0–23 months) have a higher incidence of undernutrition than older children (24–59 months) and may face a higher risk of death from undernutrition (Victora et al., 2021). Of the estimated 5.2 million child deaths recorded in 2019, 2.4 million (46%) occurred in newborns (infants under 28 days) and 1.5 million (29%) in children aged 1–11 months (World Health Organisation [WHO], 2020).

Few studies, however, have assessed the association between anthropometric deficits, age and mortality in children under five, largely due to insufficient data (Rice et al., 2000). Much of the work exploring the risk of death by age has compared the ability of different anthropometric criteria to identify children at highest risk of mortality (Garenne et al., 2019; Khara et al., 2021; O'Brien et al., 2020). Studies that have directly explored how age affects mortality risk in children 6–59 months with undernutrition, have suggested overall higher mortality for younger groups, but highlight increased mortality among older wasted children (Katz et al., 1989; Schwinger et al., 2019).

Research has also demonstrated sex differences in undernutrition whereby boys are often more likely to be wasted, stunted and underweight than girls (Costa et al., 2021; Garenne et al., 2019; Khara et al., 2018; Myatt et al., 2018; Thurstans et al., 2020). Evidence on the reasons for these differences is limited, and to date, the possible implications for treatment and mortality outcomes have not been well researched (Thurstans et al., 2022).

The aim of this analysis was to inform programming and policymaking by describing mortality risk associated with anthropometric deficits (wasting, underweight and stunting) in children 6–59 months by age and sex using multi‐country cohort data from low‐ and middle‐income countries (LMIC).

## 2 METHODS

### 2.1 Study design

This was a secondary data review and meta‐analysis of multi‐country cohort data following STROBE guidelines (Vandenbroucke et al., 2007). We assessed variations in mortality risk associated with individual anthropometric deficits (wasting, underweight and stunting), as well as whether these relationships differed by age and sex in children 6–59 months.

### 2.2 Study setting and participants

This study followed a separate analysis exploring which anthropometric criteria best identifies children at high risk of near‐term mortality (Khara et al., 2021). The same data set for 56,559 children was used for this analysis, which comprised a reduced set of variables containing basic demographic information, anthropometric measures and mortality outcomes. The data originated from 12 large, prospective community cohort studies or randomised controlled trials in LMIC. These included studies of various interventions such as vitamin A supplementation and antibiotic provision, breastfeeding and child feeding interventions and general monitoring of health and nutrition. The studies were conducted between 1977 and 2013 (Adair et al., 1993; Arifeen et al., 2001; Fawzi et al., 1997; Garenne et al., 1987; Katz et al., 1989; Martines et al., 1998; Mølbak et al., 1992; O'Brien et al., 2020; Van Den Broeck et al., 1993; West et al., 1991).

All of the original studies took place in LMIC, six in Africa (Democratic Republic of Congo, Ghana, Guinea Bissau, Niger, Senegal and Sudan), five in Asia (Bangladesh, India, Indonesia, Nepal, Philippines) and one in South America (Peru).

We focused on children aged 6–59 months old. Data were not available for children under 6 months of age in this data set.

### 2.3 Variables

The primary outcome was mortality, defined as death recorded within 6 months of a contact during which anthropometry was assessed. Mortality was confirmed by verbal autopsy in all studies, with the exception of one which examined hospital records (Mølbak et al., 1992). A contact was defined as a point in time whereby a child's anthropometric status was assessed and recorded by a health worker.

Explanatory variables were wasting (measured by weight‐for‐length/weight‐for‐height z‐score [WLZ/WHZ] or mid‐upper arm circumference [MUAC]), underweight (measured by weight‐for‐age z‐score [WAZ]) and stunting (measured by height‐for‐age z‐score [HAZ]), as well as age and sex.

We used the World Health Organisation (WHO) classifications of undernutrition for each anthropometric indicator capturing both moderate and severe cases of each deficit (World Health Organisation [WHO], 2006). Wasting was defined as WLZ/WHZ < −2, or MUAC < 125 mm. Underweight was defined as weight‐for‐age WAZ < −2 z‐score and stunting was defined as HAZ < −2 z‐score. We also conducted separate analysis for severe definitions of each deficit. Severe wasting was defined as WLZ/WHZ < −3 z‐score, or MUAC < 115 mm, severe underweight was defined as weight‐for‐age WAZ < −3 z‐score and severe stunting was defined as HAZ < −3 z‐score. Bilateral pitting oedema was not investigated as the relevant data was not present in the data set (Khara et al., 2021).

Children were stratified into two groups according to age at anthropometric assessment: younger children (aged 6–23 months) and older children (aged 24–59 months). As age was a key indicator of interest, we excluded countries where data were not available for children in both age groups. After this exclusion, eight countries remained in the data set (Democratic Republic of Congo (DRC), Guinea Bissau, Indonesia, Nepal, Niger, Philippines, Senegal and Sudan). MUAC data was only available from three countries (Senegal, Nepal and DRC).

### 2.4 Statistical methods

Z‐scores were calculated using the 2006 WHO Child Growth Standards (World Health Organisation [WHO], 2006). Records with extreme z‐score values were identified and censored using the WHO “biological plausibility” criteria (Blössner et al., 2009) We did not encounter missing data as this was a previously cleaned data set.

Statistical analysis was conducted using Stata V.16 (StataCorp 2017, Stata Statistical Software). We used the following measures to examine mortality risk among children with anthropometric deficits, conducting separate analyses for moderate and severe definitions of MUAC, WHZ, WAZ and HAZ:

1. Absolute risk of mortality/1000 within each age and sex category
$$\mathrm{Absolute}\;\mathrm{risk}\;\mathrm{mortality}/1000\;=\frac{\text{deaths}\;\mathrm{in}\;\mathrm{children}\;\mathrm{with}\;\mathrm{anthropometric}\;\mathrm{deficit}(\mathrm{age}\;\mathrm{or}\;\mathrm{sex}\;\mathrm{group})}{\mathrm{total}\;\mathrm{number}\;\mathrm{of}\;\mathrm{children}\;\mathrm{with}\;\mathrm{anthropometric}\;\mathrm{deficit}(\mathrm{age}\;\mathrm{or}\;\mathrm{sex}\;\mathrm{group})}\times 1000.$$
2. Risk ratio comparing absolute risks of mortality by age and sex categories (older versus younger children, girls versus boys).

Analysis was performed for each individual country. Significant heterogeneity was detected among the various surveys; hence a random effect model was used to take into consideration the effects of potential bias due to differences between the studies which were not due to chance.

We performed a random‐effects meta‐analysis to pool mortality risk estimates for each anthropometric deficit and compared by age and sex. Individual country and pooled effects are presented as risk ratios with 95% confidence intervals (CIs). We used the *I* ^2^ index to measure the degree of heterogeneity of effect estimates across cohorts.

### 2.5 Ethical approval

All original data was subject to the relevant ethical approval process, and permissions were sought from all original Principal Investigators (PIs) while sourcing data. This analysis has further ethical approval from the London School of Hygiene and Tropical Medicine ethics committee (Reference 22958).

## 3 RESULTS

### 3.1 Study characteristics

**Figure 1:**
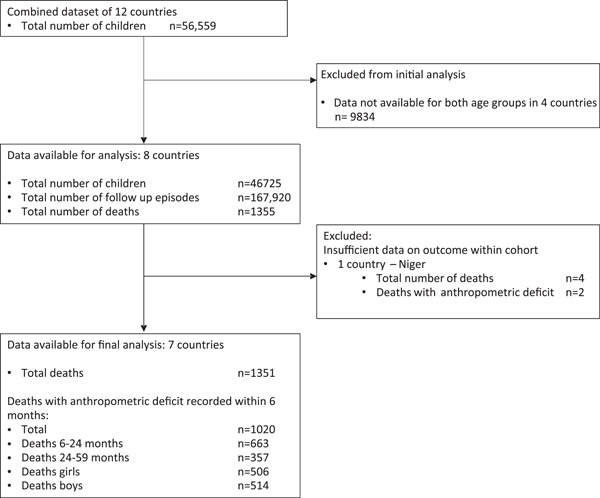
Study flow chart.

Figure 1 shows the study flow diagram. After initial analysis we excluded Niger due to rarity of deaths (*n*= 4, two of which had anthropometric deficits recorded), resulting in insufficient power in this cohort following disaggregation.

Characteristics of the studies in the final analysis are presented in Table 1. The seven‐country data set comprised 45,755 children, inclusive of 22,325 girls (48.8%) and 23,430 boys (51.2%). The age categories included 19,785 (43.2%) children aged 6–23 months and 25,970 (56.8%) children aged 24–59 months. A total of 166,755 follow‐up contacts were recorded.

**Table 1:** Study characteristics table

Country	Study	Recruitment years	Study intervention	Children aged 6–59 months	No follow‐up episodes	Duration of anthropometric follow‐up in months Median (Range)	Loss to follow‐up (%)	Total deaths (with or without anthropometric deficit)	Deaths (girls) *N* (%)	Deaths (boys) *N* (%)	Deaths 6–23 m age group *N* (%)	Deaths 24–59 m age group *N* (%)
DRC	Van Den Broeck (1993)	1989–1993	Longitudinal health and nutrition monitoring	4584	17,918	11 [0–10]	No info	196	91 (46.4)	105 (53.6)	122 (62.2)	74 (38.8)
Guinea‐Bissau	Mølbak (1992)	1987–1990	Child mortality audit	985	4,385	11 [0–37]	No info	118	66 (55.9)	52 (44.1)	86 (72.9)	32 (27.1)
Indonesia	Katz (1989)	1977	Longitudinal nutrition monitoring	3806	17,367	15 [0–18]	7.8%	214	104 (48.6)	110 (51.4)	121 (56.5)	93 (43.5)
Nepal	West (1991)	1989	Vitamin A RCT	5883	25,159	16 [0–25]	6%	128	70 (54.7)	58(45.3)	73 (57.0)	55 (43.0%)
Philippines	Adair (1993)	1982–1983	Longitudinal Health and nutrition survey	2823	25,031	18 [0–19]	11.9%	233	99 (42.5)	134 (57.5)	233 (100)	0 (0)
Senegal	Garenne (1987)	1983	Longitudinal nutrition survey	5142	12,615	11 [0–19]	12.2%	327	166 (50.8)	161 (49.2)	196 (59.9)	131 (40.1)
Sudan	Fawzi (1997)	1988	Longitudinal nutrition survey/Vitamin A RCT	22532	64,280	12 [0–19]	16%	135	82 (60.7)	53 (39.3)	75 (55.6)	60 (44.4)
Total				45,755	166,755			1,351	678 (50.2)	673 (49.8)	906 (67.1)	445 (32.9)

Overall, 1351 deaths were recorded. We present a breakdown of deaths by anthropometric deficit in Tables 2 and S2a. Of the total deaths, 1020 (75.6%) occurred in children with anthropometric deficits. Among the deaths, a total of 506 (49.6%) were for girls and 514 (50.4%) for boys. There were more deaths recorded within 6 months of an anthropometric deficit in children aged 6–23 months compared with those 24–59 months of age (*n* = 663 [65%] versus *n* = 357 [35%], respectively) (see Figure 1).

**Table 2:** Child mortality (deaths within 6 months) by anthropometric deficit according to geographic location, age and sex – moderate

		6–23 months				24–59 months			
		Male		Female		Male		Female	
Country	Anthropometric indicator	*n* died/*n* with deficit	%	*n* died/*n* with deficit	%	*n* died/*n* with deficit	%	*n* died/*n* with deficit	%
DRC	MUAC < 125 mm	40/1341	3.0	33/1589	2.1	13/1162	1.1	16/1127	1.4
	WHZ < −2	10/251	4.0	5/169	3.0	4/184	2.2	7/116	6.0
	WAZ < −2	34/1057	3.2	19/767	2.5	23/2055	1.1	20/1735	1.2
	HAZ < −2	47/1870	2.5	24/1487	1,6	29/4504	0.6	30/3734	0.8
Guinea‐Bissau	WHZ < −2	5/100	5.0	4/60	6.7	3/39	7.7	2/28	7.1
	WAZ < −2	13/243	5.4	15/211	7.1	4/108	3.7	7/196	3.6
	HAZ < −2	21/407	5.2	26/396	6.6	9/435	2.1	11/387	2.8
Indonesia	WHZ < −2	10/249	4.0	5/160	3.1	7/221	3.2	7/120	5.8
	WAZ < −2	37/859	4.3	25/616	4.1	26/2138	1.2	29/1935	1.5
	HAZ < −2	46/1461	3.2	31/1124	2.8	34/4124	0.8	46/3697	1.2
Nepal	MUAC < 125 mm	9/951	1.0	38/1566	2.4	10/325	3.1	5/482	1.0
	WHZ < −2	10/929	1.1	26/879	3.0	9/665	1.4	5/462	1.1
	WAZ < −2	19/2334	0.8	39/2202	1.8	25/4268	0.6	16/4207	0.4
	HAZ < −2	20/2812	0.7	33/2605	1.3	28/6364	0.4	23/6043	0.4
Philippines	WHZ < −2	55/1242	4.4	32/897	3.6	0/61	0.0	0/45	0.0
	WAZ < −2	87/3779	2.3	72/3072	2.3	0/379	0.0	0/374	0.0
	HAZ < −2	89/5894	1.5	74/4454	1.7	0/686	0.0	0/596	0.0
Senegal	MUAC < 125 mm	29/377	7.7	26/417	6.2	16/141	11.4	14/143	9.8
	WHZ < −2	47/665	7.1	35/533	6.6	28/394	7.1	22/438	5.0
	WAZ < −2	63/1134	5.6	57/1029	5.5	42/936	4.5	38/905	4.2
	HAZ < −2	32/562	5.7	25/378	6.6	37/1122	3.3	38/961	4.0
Sudan	WHZ < −2	12/827	1.5	19/576	3.3	8/2489	0.3	10/1767	0.6
	WAZ < −2	15/2013	0.8	30/1625	1.9	20/10,023	0.2	19/10,169	0.2
	HAZ < −2	16/2838	0.6	29/2320	1.3	19/13,849	0.1	19/13,493	0.1
Total	MUAC < 125 mm	78/2689	2.9	97/3597	2.7	39/1642	2.4	35/1767	2.0
	WHZ < −2	149/4296	3.5	126/3300	3.8	59/4089	1.4	53/3002	1.8
	WAZ < −2	268/11,486	2.3	257/9585	2.7	142/20,199	0.7	129/19,775	0.7
	HAZ < −2	271/15,925	1.7	242/12,830	1.9	158/31,473	0.5	167/29,233	0.6

### 3.2 Wasting measured by MUAC

Three country cohorts were included in the analysis for MUAC (Table 3 and Figure 2). We compared absolute risk of mortality in younger children with absolute risk of mortality in older children with MUAC < 125 mm. In two of the three cohorts, absolute risk of death was higher in younger children, with evidence of a difference between age groups in one cohort (DRC). In the remaining cohort (Senegal), the risk was higher for older children with borderline evidence of a difference between age groups. After meta‐analysis, the combined effect size was RR 1.08 (95% CI 0.53–2.22, *p* = 0.826), suggesting no difference in absolute risk of death between older and younger children with MUAC < 125 mm. Our results were similar when the same analysis was performed for MUAC < 115 mm, with no observed difference in the risk of death between younger and older children (RR 0.77, 95% CI 0.47–1.26, *p* = 0.302; Table S4).

**Table 3:** Absolute risk (AR) of mortality per 1000 children within 6 months of a contact and associated anthropometric deficits by age and relative risk (RR) of mortality in younger compared with older children

	**MUAC** < **125 mm**														
	**Both sexes**					**Girls**					**Boys**				
	**AR 6–23 m**	**AR 24–59 m**	**RR 6–23 m versus 24‐59 m (ref)**	**95% CI**	***p***	**AR 6–23 m**	**AR 24–59 m**	**RR 6–23 m versus 24–59 m (ref)**	**95% CI**	***p***	**AR 6–23 m**	**AR 24–59 m**	**RR 6–23 m versus 24–59 m (ref)**	**95% CI**	***p***
DRC	24.91	12.67	1.96	1.28–3.01	0.002	20.76	14.19	1.46	0.81–2.64	0.205	29.82	11.18	2.66	1.43–4.96	0.001
Nepal	18.67	18.58	1	0.56–1.79	0.988	24.26	10.37	2.33	0.93–5.91	0.063	9.46	30.76	0.3	0.13–0.75	0.006
Senegal	69.26	105.63	0.65	0.43–1.00	0.051	62.35	97.9	0.63	0.34–1.19	0.154	76.92	113.47	0.67	0.38–1.21	0.189
Pooled estimate			**1.08**	**0.53–2.22**	**0.826**			**1.28**	**0.59–2.77**	**0.530**			**0.71**	**0.30–1.71**	**0.447**

	**WHZ** < **−2**														
	**Both sexes**					**Girls**					**Boys**				
	**AR 6–23 m**	**AR 24–59 m**	**RR 6–23 m versus 24‐59 m (ref)**	**95% CI**	***p***	**AR 6–23 m**	**AR 24–59 m**	**RR 6–23 m versus 24–59 m (ref)**	**95% CI**	***p***	**AR 6–23 m**	**AR 24–59 m**	**RR 6–23 m versus 24–59 m (ref)**	**95% CI**	***p***
DRC	35.71	36.66	0.97	0.45–2.09	0.946	29.58	60.34	0.49	0.16–1.51	0.204	39.84	21.73	1.83	0.58–5.75	0.291
Guinea Bissau	56.25	74.62	0.75	0.26–2.17	0.600	66.66	71.42	0.93	0.18–4.80	0.934	50	76.92	0.65	0.16–2.59	0.540
Indonesia	36.67	41.05	0.89	0.44–1.82	0.757	31.25	58.33	0.53	0.17–1.65	0.268	40.16	31.67	1.26	0.49–3.27	0.623
Nepal	19.91	12.42	1.6	0.87–2.96	0.127	29.57	10.82	2.73	1.06–7.07	0.030	10.76	13.53	0.79	0.32–1.95	0.615
Philippines	40.67	0				35.67	0				44.28	0			
Senegal	68.44	60.09	1.13	0.81–1.60	0.453	65.66	50.22	1.3	0.78–2.19	0.309	70.67	71.06	0.99	0.63–1.56	0.981
Sudan	22.09	4.22	5.23	2.93–9.31	<0.001	32.98	5.65	5.83	2.73–12.46	<0.001	14.51	3.21	4.52	1.85–11.01	<0.001
Pooled estimate			**1.35**	**0.79–2.33**	**0.272**			**1.32**	**0.66–2.65**	**0.430**			**1.32**	**0.83; 2.12**	**0.245**

	**WAZ** < **−2**														
	**Both sexes**					**Girls**					**Boys**				
	**AR 6–23 m**	**AR 24–59 m**	**RR 6–23 m versus 24‐59 m (ref)**	**95% CI**	***p***	**AR 6–23 m**	**AR 24–59 m**	**RR 6–23 m versus 24–59 m (ref)**	**95% CI**	***p***	**AR 6–23 m**	**AR 24–59 m**	**RR 6–23 m versus 24–59 m (ref)**	**95% CI**	***p***
DRC	29.05	11.34	2.56	1.72–3.81	<0.001	24.77	11.52	2.15	1.15–4.00	0.014	32.16	11.19	2.87	1.70–4.85	<0.001
Guinea Bissau	61.67	36.18	1.7	0.86–3.37	0.119	71.09	35.71	1.99	0.83–4.78	0.115	53.49	37.03	1.44	0.48–4.33	0.507
Indonesia	42.03	13.5	3.11	2.18–4.45	<0.001	40.58	14.98	2.7	1.60–4.59	<0.001	43.07	12.16	3.54	2.16–5.81	<0.001
Nepal	12.78	4.83	2.64	1.77–3.93	<0.001	17.71	3.8	4.66	2.61–8.31	<0.001	8.14	5.85	1.39	0.77–2.52	0.276
Philippines	23.2	0				23.43	0				23.02	0			
Senegal	55.47	43.45	1.27	0.97–1.68	0.082	55.39	41.98	1.31	0.88–1.97	0.174	55.55	44.87	1.23	0.85–1.81	0.270
Sudan	12.36	1.93	6.4	4.18–9.82	<0.001	18.46	1.86	9.92	5.58–17.51	<0.001	7.45	1.99	3.74	1.92–7.28	<0.001
Pooled estimate			**2.57**	**1.65–4.00**	**<0.001**			**2.97**	**1.65–5.36**	**<0.001**			**2.13**	**1.40–3.24**	**<0.001**

	**HAZ** < **−2**														
	**Both sexes**					**Girls**					**Boys**				
	**AR 6–23 m**	**AR 24–59 m**	**RR 6–23 m versus 24‐59 m (ref)**	**95% CI**	***p***	**AR 6–23 m**	**AR 24–59 m**	**RR 6–23 m versus 24‐59 m (ref)**	**95% CI**	***p***	**AR 6–23 m**	**AR 24–59 m**	**RR 6–23 m versus 24‐59 m (ref)**	**95% CI**	***p***
DRC	21.14	7.16	2.95	2.09–4.16	<0.001	16.13	8.03	2	1.18–3.42	0.009	25.13	6.43	3.9	2.47–6.18	<0.001
Guinea Bissau	58.53	24.33	2.4	1.44–4.02	0.001	65.65	28.42	2.3	1.16–4.61	0.014	51.59	20.68	2.49	1.16–5.38	0.016
Indonesia	29.78	10.22	2.91	2.14–3.97	<0.001	27.58	12.44	2.21	1.41–3.48	<0.001	31.48	8.24	3.82	2.46–5.93	<0.001
Nepal	9.78	4.11	2.37	1.62–3.49	<0.001	12.66	3.8	3.33	1.96–5.66	<0.001	7.11	4.39	1.61	0.91–2.86	0.097
Philippines	15.75	0				16.61	0				15.1	0			
Senegal	60.63	36	1.68	1.20–2.36	0.002	66.13	39.54	1.67	1.02–2.73	0.039	56.93	32.97	1.72	1.09–2.74	0.019
Sudan	8.72	1.38	6.31	4.08–9.66	<0.001	12.5	1.4	8.92	4.99–15.80	<0.001	5.63	1.37	4.1	2.12–7.98	<0.001
Pooled estimate			**2.83**	**2.09; 3.82**	**<0.001**			**2.82**	**1.87–4.24**	**<0.001**			**2.75**	**1.97–3.85**	**<0.001**

*Note*: AR represents the absolute risk of death in the exposed group per 1000 children; RR represents the relative risk of death in young (6–23 months) versus older (24–59 months; reference group) age group; pooled estimate represents the weighted pooled estimates from the meta‐analysis.

Figure 2Forest plots for pooled risk ratios of mortality in children 6–23 months versus 24–59 months for MUAC < 125 mm WHZ < −2, WAZ < −2 and HAZ < −2. (a) Mortality risk ratio between younger and older (reference group) age group for MUAC < 125 mm. (b) Mortality risk ratio between younger and older (reference group) age group for WHZ < −2. (c) Mortality risk ratio between younger and older (reference group) age group for WAZ < −2. (d) Mortality risk ratio between younger and older (reference group) age group for HAZ < −2. Estimates on the left part of the axis suggest a higher mortality in older children, and estimates on the right part of the axis suggest a higher mortality among younger children.

**Figure:**
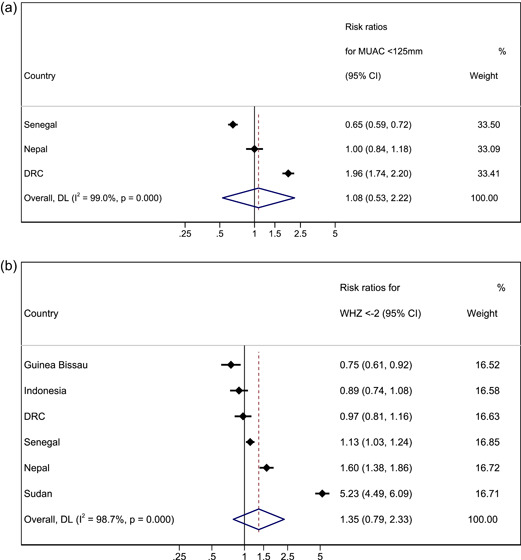

**Figure:**
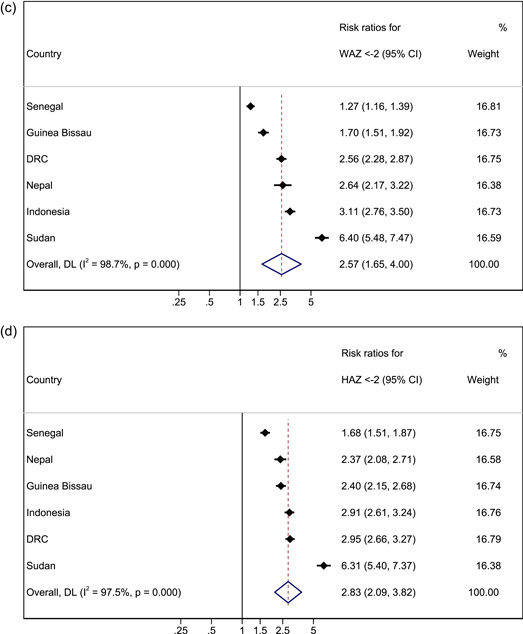

When assessing the risk of death for boys versus girls (reference group) in all age groups with a MUAC < 125 mm in a pooled meta‐analysis, we did not observe differences in the risk of death in either the younger age group or the older age group (RR 0.93, 95% CI 0.46–1.86, *p* = 0.838 and RR 1.27, 95% CI 0.65–2.45, *p* = 0.484 respectively; Table S3a). One exception to this was Nepal, where younger boys had a lower relative risk of death than younger girls (RR 0.39, 95% CI 0.19–0.80, *p* = 0.008), but older boys with MUAC < 125 mm had a higher risk of mortality than older girls with MUAC < 125 mm (RR 2.97, 95% CI 1.02–8.60, *p* = 0.035).

### 3.3 Wasting measured by WHZ

Six country cohorts were included in the analysis for WHZ < −2 as no deaths were recorded for this deficit in the older age group in the Philippines (Table 3 and Figure 2). In three of the cohorts, absolute risk of mortality was higher in younger children, with evidence of a difference in one cohort (Sudan). In the other three cohorts, absolute risk was higher in older children but not significantly so. The pooled meta‐analysis found no significant difference in absolute mortality risk in younger compared with older children (RR 1.35, 95% CI 0.79–2.33, *p* = 0.272). Our results were similar when the meta‐analysis was performed for WHZ < −3, with no observed difference in the absolute risk of death between younger and older children (RR 1.21, 95% CI 0.66–2.22, *p* = 0.540; Table S4).

Overall, after meta‐analysis we did not observe a significant difference in absolute mortality risk between boys and girls (reference group) in either the younger or the older age group (RR 0.83, 95% CI 0.55–1.26, *p* = 0.388 and RR 0.84, 95% CI 0.52–1.36, *p* = 0.478 respectively). The exceptions were in Nepal and Sudan, where younger boys with WHZ < −2 had a lower absolute risk of death than younger girls with the same anthropometric deficit (RR 0.36; CI 0.18–0.75 *p* = 0.004, and RR 0.44; 95% CI 0.21–0.90, *p* = 0.021 respectively).

### 3.4 Underweight

All seven country cohorts were included in our analysis of WAZ < −2 (Table 3 and Figure 2). Our results showed consistently that younger underweight children have a higher absolute risk of death within 6 months of measurements than older children in all cohorts, significantly so in Nepal and Sudan. After meta‐analysis, our combined effect size was RR 2.57, (95% CI 1.65–4.00, *p* < 0.001). Results from the analysis are presented in Table 3 and Figure 2). Our results were similar when meta‐analysis was performed for WAZ < −3, whereby younger children had a higher absolute risk of death when compared with older children (RR 2.05, 95% CI 1.13–3.73, *p* = 0.018; Table S4).

For sex, the pooled meta‐analysis results for underweight children showed no significant difference in the absolute risk of death between girls and boys in younger and older age groups (RR 0.82, 95% CI 0.61–1.09, *p* = 0.176 and RR 1.05 95% CI 0.82–1.33, *p* = 0.708, respectively). However, we did observe a lower risk of death for younger boys compared with younger girls in Nepal (WAZ, RR 0.46, 95% CI 0.27–0.79, *p* = 0.004).

### 3.5 Stunting

Seven country cohorts were included in our analysis of HAZ < −2 (Table 3 and Figure 2). Our results showed consistently that stunted younger children had a higher absolute risk of death within 6 months of measurements than older children, significantly so in Nepal and Sudan. After meta‐analysis, the combined effect size for HAZ was RR 2.83 (95% CI 2.09–3.82, *p* < 0.001). Similarly, when meta‐analysis was performed for HAZ < −3, younger children had a significantly higher absolute risk of death when compared with older children (RR 2.74, 95% CI 1.74–4.32, *p* < 0.001; Table S4).

For sex, the pooled meta‐analysis showed no significant difference in the risk of death between stunted girls and boys in both younger and older age groups (RR 0.88, 95% CI 0.67–1.14, *p* = 0.318 and RR 0.82, 95% CI 0.66–1.02, *p* = 0.070, respectively). However, we did observe a lower risk of death for younger boys compared with younger girls in Nepal (HAZ RR 0.56, 95% CI 0.32–0.98, *p* = 0.038) and for younger boys with HAZ < −2 compared with younger girls with HAZ < −2 in Sudan (RR 0.51, 95% CI 0.25–0.83, *p* = 0.008).

Using the *I* ^2^ index from the meta‐analysis results (Figures 2 and 3), we found strong evidence of heterogeneity, which was not explained by age and sex. This suggests pooled estimates should be interpreted with caution as true differences in effect are likely due to influences not measured or adjusted for in this analysis.

Figure 3Forest plots for pooled risk ratios of absolute risk in children 6–23 months versus 24–59 months by sex for MUAC, WHZ, WAZ and HAZ. (a) Mortality risk ratio between younger boys and girls (reference group) for MUAC < 125 mm. (b) Mortality risk ratio between older boys and girls (reference group) for MUAC < 125 mm. (c) Mortality risk ratio between younger boys and girls (reference group) for WHZ < −2. (d) Mortality risk ratio between older boys and girls (reference group) for WHZ < −2. (e) Mortality risk ratio between younger boys and girls (reference group) for WAZ < −2. (f) Mortality risk ratio between older boys and girls (reference group) for WAZ < −2. (g) Mortality risk ratio between younger boys and girls (reference group) for HAZ < −2. (h) Mortality risk ratio between older boys and girls (reference group) for HAZ < −2. Estimates on the left part of the axis suggest a higher mortality in girls, and estimates on the right part of the axis suggest a higher mortality among boys.

**Figure:**
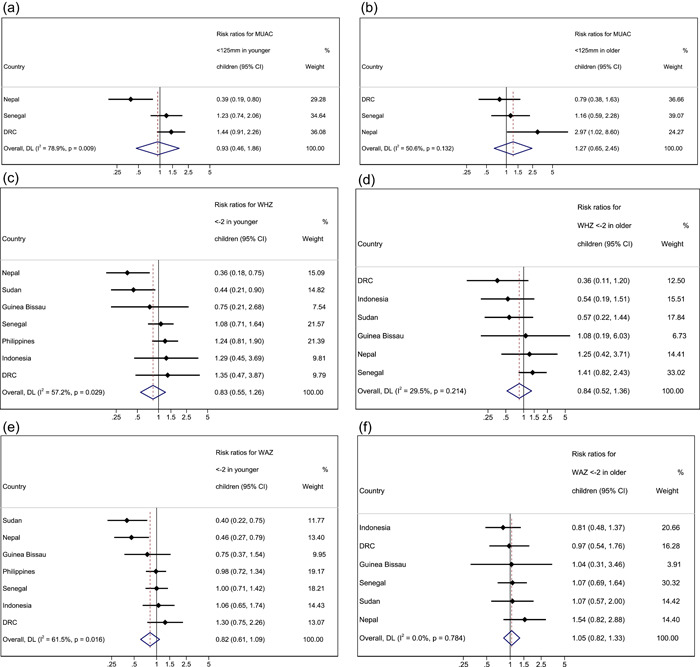

**Figure:**
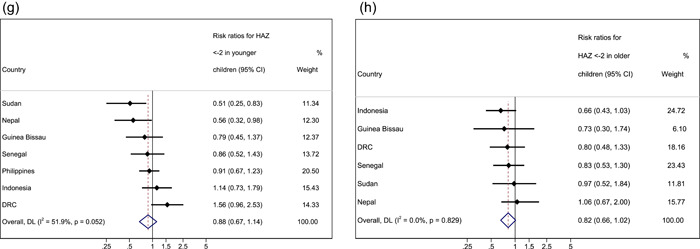

## 4 DISCUSSION

This analysis aimed to evaluate mortality risk associated with anthropometric deficits in children aged 6–59 months by age and sex. Our findings suggest that in wasted children, as measured by MUAC or WHZ, there is no significant difference in absolute mortality risk between older and younger age groups. For underweight and stunting, the absolute mortality risk is higher in younger compared with older children. Our findings were similar when the analysis was repeated for severe deficits. In terms of sex, our results suggest that girls and boys have a similar absolute mortality risk associated with each of the four anthropometric deficits, regardless of age.

Wasting is known to be associated with high mortality (McDonald et al., 2013) and is more common in younger than older children (Karlsson et al., 2022; Mertens et al., 2020). Here, we similarly found higher numbers of wasted children under 2 years old and a higher proportion of deaths in that age group compared with children older than 2 years. However, there were no differences in mortality risk between age groups. This suggests that, while a higher proportion of younger children may be targeted by wasting treatment programmes, older children with anthropometric deficits are similarly vulnerable to mortality and should not be neglected.

Some previous studies have suggested that the risk of death from wasting (as measured by WHZ) might be higher in older children. A multi‐country pooled analysis (DRC, Senegal and Nepal) of children aged 6–59 months found hazard ratios for children with wasting (WHZ) to be higher in older children (≥2 years), though not significantly so [10]. This was also reported in a study from Indonesia, whereby moderate to severe wasting was associated with increased mortality risk, more so in older children than in younger children [20]. However, the sample sizes in this study were very small and statistical testing was not reported. Future research looking at z‐scores and age as continuous rather than binary variables might help to clarify the association between age and mortality risk associated with wasting.

Overall our results showed a higher proportion of deaths in younger children compared with older children, a finding consistent with previous research (Pelletier et al., 1994). We found higher absolute mortality risk for younger children who are underweight or stunted. This suggests that, in resource‐limited settings, programmes which use these measures to target nutrition interventions may be justified in prioritising younger children. WAZ is increasingly recognised as a composite indicator of multiple anthropometric deficits and increased mortality risk (McDonald et al., 2013; Myatt et al., 2018). Evidence shows that the peak incidence of both wasting and stunting is between 0–3 months (Mertens et al., 2020), so early interventions to prevent the accumulation of anthropometric deficits (Thurstans et al., 2021) are essential, especially with the greater risk of mortality from being stunted or underweight in younger children. Evidence around the importance of meeting nutrition requirements in the first 1000 days, and the presence of wasting and stunting at birth, supports extension of nutrition programming to include the preconception and prenatal periods (Victora et al., 2021).

In relation to sex, while studies have shown that boys are more likely to be wasted, stunted and underweight than girls (Khara et al., 2018; Myatt et al., 2018; Myatt et al., 2019; Odei Obeng‐Amoako et al., 2020; Thurstans et al., 2020). Our findings suggest that mortality risk is similar between the sexes. Studies of diarrhoeal disease in children aged between 12 and 59 months have found similar results, indicating that despite slightly higher incidence rates for boys, cause‐specific mortality is higher amongst girls, perhaps due to health‐seeking behaviours such as later presentation to professional health settings or later provision of ORS for girls (World Health Organisation [WHO], 2007). Despite there being no difference in the relative risk of mortality between boys and girls, the greater number of boys affected by wasting, stunting and underweight, suggests that greater numbers of boys will die of undernutrition than girls in absolute terms. In our study, we did observe a difference in Nepal, whereby girls had a significantly higher risk of death than boys for each of the anthropometric deficits. Previous research has suggested that sex differences in undernutrition might be age and context‐specific (Costa et al., 2021; Thurstans et al., 2020) and influenced by environmental and social factors. For example, the disadvantage in linear growth for boys is most evident in the first years, but by the age of 4 years, the sex gap has mostly disappeared, and in some countries, the gap has been reversed (Costa et al., 2021). Programme data should be analysed by both age and sex to understand geographic, environmental, and social context‐specific differences in growth‐failure‐associated mortality risk.

### 4.1 Strengths and limitations

One of the key strengths of this study is the unique nature of the data. We analysed community cohorts with information recorded on anthropometric indices and mortality and were able to pool cohort data from multiple countries. The large sample sizes provided by this approach enabled us to examine mortality risk by age and sex, as mortality is a rare outcome in individual cohorts. However, we do recognise some limitations.

The first is the absence of data for infants under 6 months, likely resulting in an underestimation of the impacts of anthropometric deficits in children under two. There is increased recognition of the importance of including infants under 6 months within nutrition programmes and surveys, alongside evidence that undernutrition often occurs before 6 months and is associated with high mortality (Mwangome et al., 2017; Victora et al., 2021). Though this is a clear limitation, our findings contribute to the evidence base for increased vulnerability before age two.

Second, there is potential for the introduction of bias from loss to follow‐up in the original studies (see Table 1), leading to survivor bias if deaths were higher amongst those lost to follow‐up. It was not possible to quantify this from the original studies (Khara et al., 2021). The age of the cohorts might also be a factor to consider. Much has changed since the data was collected on these cohorts, especially with respect to the availability of programmes targeting these age groups, which limits the generalisability of these results.

A further limitation is that we did not have data on potential confounders such as, socioeconomic status, health indicators such as diarrhoea, HIV, respiratory illnesses, breastfeeding status, complementary feeding, and care practices, or seasonal indicators. It was therefore not possible to explain the heterogeneity between studies or elucidate on contextual differences that might directly or indirectly influence the relationships between anthropometric deficits and mortality risk. Similarly, two of the datasets (Sudan and Nepal) were from RCTs of vitamin A supplementation and we could not adjust for the treatment group in the analysis of these datasets. In the Sudan trial, vitamin A supplementation did not have an effect on child growth or mortality (Fawzi et al., 1997; Herrera et al., 1992); therefore, it is unlikely that this variable would influence the association between anthropometric deficits and mortality in a substantial way. However, in the Nepal trial (West et al., 1991), vitamin A significantly reduced the risk of mortality; thus, the exclusion of this variable from our analysis of this data set may have led to relative risk estimates that underestimate the risk between anthropometric deficits and mortality. Some previous analysis of these data highlighted how possible access to nutrition rehabilitation and broad‐spectrum antimicrobial treatment in Niger might have protected against risk of death and might in turn explain the low number of deaths observed in this cohort (Khara et al., 2021). Further research which controls for study effects and allows for consideration of other potential explanatory factors including multiple anthropometric deficits alongside age and sex would be useful to identify any differences in results. Finally, data was only available for MUAC from 3 countries. This means a smaller sample size was available for these analyses with potentially less power to detect differences and reduced generalisability of results.

## 5 CONCLUSION

Our findings demonstrate that for wasted children there is no difference in mortality risk between younger and older children, This is also true for severely wasted children. This supports the continued inclusion of all high‐risk children under five in wasting treatment programmes. The risk of mortality associated with underweight, and stunting is higher among younger children. Again, this is also true for severe stunting and underweight. This suggests that nutrition prevention programmes might be justified in focusing limited resources on younger children. There does not appear to be a difference in mortality risk between girls and boys for any anthropometric deficit, suggesting no need to adjust current approaches according to sex.

## AUTHOR CONTRIBUTIONS

Susan Thurstans, Martha Mwangome, André Briend, Tanya Khara, Stephanie V. Wrottesley and Bridget Fenn designed the study. Data was contributed by Michel Garenne, Christine M. McDonald, Robert E. Black, Keith P. West, and Sunita Taneja. Analysis and interpretation was conducted by Stephanie V. Wrottesley, Bridget Fenn, and Susan Thurstans, discussed with and reviewed by all authors. Susan Thurstans wrote the paper. All authors have read and approved the final manuscript.

## CONFLICTS OF INTEREST

The authors declare no conflicts of interest.

## Supporting information

Supporting information.

## Data Availability

Data sharing is not applicable to this article as no new data were created or analysed in this study. Data from previously published studies was sourced directly from original investigators as listed in the manuscript for this specific secondary analysis.
