# Supplementary material for: Anthropometric deficits and the associated risk of death by age and sex in children aged 6–59 months: A meta‐analysis
Source: Matern Child Nutr. 2022 Sep 27;19(1):e13431. doi: 10.1111/mcn.13431 (PMC9749608; doi:10.1111/mcn.13431)
Supplement: Supplementary file 1 — Supporting information. [file MCN-19-e13431-s001.docx]

**Supplementary Table 2a: Child mortality (deaths within 6 months) by anthropometric deficit according to geographic location, age and sex – severe**

|  |  | **6-23 months** | | | | **24-59 months** | | | |
| --- | --- | --- | --- | --- | --- | --- | --- | --- | --- |
|  |  | **Male** | | **Female** | | **Male** | | **Female** | |
| **Country** | **Anthropometric indicator** | **N** | **%** | **N** | **%** | **N** | **%** | **N** | **%** |
| ***DRC*** | MUAC <115 mm | 16/390 | 4.1 | 16/543 | 3.0 | 7/246 | 2.9 | 10/269 | 3.7 |
|  | WHZ <-3 | 6/45 | 13.3 | 1/28 | 3.6 | 1/25 | 4.0 | 2/20 | 10.0 |
|  | WAZ <-3 | 15/330 | 4.6 | 7/198 | 3.5 | 11/581 | 1.9 | 12/476 | 2.5 |
|  | HAZ <-3 | 29/912 | 3.2 | 9/523 | 1.7 | 24/2,277 | 1.1 | 20/1,749 | 1.1 |
| ***Guinea-Bissau*** | WHZ <-3 | 4/19 | 21.1 | 2/12 | 16.7 | 1/6 | 16.7 | 0/6 | 0.0 |
|  | WAZ <-3 | 7/81 | 8.6 | 5/57 | 8.8 | 3/31 | 9.7 | 2/44 | 4.6 |
|  | HAZ <-3 | 9/148 | 6.1 | 12/122 | 9.8 | 4/105 | 3.8 | 4/129 | 3.1 |
| ***Indonesia*** | WHZ <-3 | 3/54 | 5.6 | 1/26 | 3.9 | 1/31 | 3.2 | 4/20 | 20.0 |
|  | WAZ <-3 | 13/242 | 5.4 | 7/172 | 4.1 | 16/546 | 2.9 | 13/515 | 2.5 |
|  | HAZ <-3 | 31/616 | 5.0 | 19/433 | 4.4 | 22/2,115 | 1.0 | 30/1,852 | 1.6 |
| ***Nepal*** | MUAC <115 mm | 3/211 | 1.4 | 17/463 | 3.7 | 5/35 | 14.3 | 2/65 | 3.1 |
|  | WHZ <-3 | 3/231 | 1.3 | 8/201 | 4.0 | 4/78 | 5.1 | 1/54 | 1.9 |
|  | WAZ <-3 | 11/994 | 1.1 | 29/954 | 3.0 | 14/1,220 | 1.2 | 7/1,428 | 0.5 |
|  | HAZ <-3 | 11/1,497 | 0.7 | 24/1,211 | 2.0 | 23/3,354 | 0.7 | 15/3,318 | 0.5 |
| ***Philippines*** | WHZ <-3 | 31/273 | 11.4 | 15/144 | 10.4 | 0/9 | 0.0 | 0/4 | 0.0 |
|  | WAZ <-3 | 55/1,058 | 5.2 | 38/819 | 4.6 | 0/102 | 0.0 | 0/90 | 0.0 |
|  | HAZ <-3 | 48/2,412 | 2.0 | 36/1,619 | 2.2 | 0/325 | 0.0 | 0/290 | 0.0 |
| ***Senegal*** | MUAC <115 mm | 10/102 | 9.8 | 11/85 | 12.9 | 5/29 | 17.2 | 5/46 | 10.9 |
|  | WHZ <-3 | 12/158 | 7.6 | 13/103 | 12.6 | 6/68 | 8.8 | 9/78 | 11.5 |
|  | WAZ <-3 | 30/426 | 7.0 | 29/366 | 7.9 | 20/236 | 8.5 | 18/248 | 7.3 |
|  | HAZ <-3 | 8/157 | 5.1 | 8/78 | 10.3 | 23/408 | 5.6 | 18/311 | 5.8 |
| ***Sudan*** | WHZ <-3 | 8/229 | 3.5 | 10/129 | 7.8 | 6/437 | 1.4 | 5/246 | 2.0 |
|  | WAZ <-3 | 13/731 | 1.8 | 19/569 | 3.3 | 11/3,275 | 0.3 | 14/3,223 | 0.4 |
|  | HAZ <-3 | 10/1,337 | 0.8 | 22/1,018 | 2.2 | 11/6,698 | 0.2 | 14/6,582 | 0.2 |
| ***Total*** | MUAC <115 mm | 29/707 | 4.1 | 44/1,096 | 4.0 | 17/312 | 5.5 | 17/381 | 4.5 |
|  | WHZ <-3 | 67/1,019 | 6.6 | 50/650 | 7.7 | 19/660 | 2.9 | 21/431 | 4.9 |
|  | WAZ <-3 | 144/3,895 | 3.7 | 134/3,164 | 4.2 | 76/6,104 | 1.3 | 66/6,144 | 1.1 |
|  | HAZ <-3 | 146/7,126 | 2.1 | 130/5,040 | 2.6 | 109/15,555 | 0.7 | 101/14,460 | 0.7 |

**Supplementary table 3a: Absolute risk of mortality associated with anthropometric deficits by sex and relative risk of mortality in younger compared to older children.**

|  | **MUAC <125mm** | | | | | | | | | |
| --- | --- | --- | --- | --- | --- | --- | --- | --- | --- | --- |
|  | ***AR 6-23m*** | | ***RR boys vs. girls (ref)*** | | | ***AR 24-59m*** | | ***RR boys vs. girls (ref)*** | | |
|  | ***Girls*** | ***Boys*** | ***β*** | ***95% CI*** | ***P*** | ***Girls*** | ***Boys*** | ***β*** | ***95% CI*** | ***P*** |
| ***DRC*** | 20.76 | 29.82 | 1.44 | 0.91; 2.26 | 0.117 | 14.19 | 11.18 | 0.79 | 0.38; 1.63 | 0.520 |
| ***Nepal*** | 24.26 | 9.46 | 0.39 | 0.19; 0.80 | 0.008 | 10.37 | 30.76 | 2.97 | 1.02; 8.60 | 0.035 |
| ***Senegal*** | 62.35 | 76.92 | 1.23 | 0.74; 2.06 | 0.419 | 97.9 | 113.47 | 1.16 | 0.59; 2.28 | 0.669 |
| ***Pooled estimate*** |  |  | **0.93** | **0.46; 1.86** | **0.838** |  |  | **1.27** | **0.65; 2.45** | **0.484** |
|  | **WHZ <-2** | | | | | | | | | |
|  | ***AR 6-23m*** | | ***RR boys vs. girls (ref)*** | | | ***AR 24-59m*** | | ***RR boys vs. girls (ref)*** | | |
|  | ***Girls*** | ***Boys*** | ***β*** | ***95% CI*** | ***P*** | ***Girls*** | ***Boys*** | ***β*** | ***95% CI*** | ***P*** |
| ***DRC*** | 29.58 | 39.84 | 1.35 | 0.47; 3.87 | 0.579 | 60.34 | 21.73 | 0.36 | 0.11; 1.20 | 0.083 |
| ***Guinea Bissau*** | 66.66 | 50 | 0.75 | 0.21; 2.68 | 0.658 | 71.42 | 76.92 | 1.08 | 0.19; 6.03 | 0.933 |
| ***Indonesia*** | 31.25 | 40.16 | 1.29 | 0.45; 3.69 | 0.640 | 58.33 | 31.67 | 0.54 | 0.19; 1.51 | 0.236 |
| ***Nepal*** | 29.57 | 10.76 | 0.36 | 0.18; 0.75 | 0.004 | 10.82 | 13.53 | 1.25 | 0.42; 3.71 | 0.686 |
| ***Philippines*** | 35.67 | 44.28 | 1.24 | 0.81; 1.90 | 0.320 | 0 | 0 |  |  |  |
| ***Senegal*** | 65.66 | 70.67 | 1.08 | 0.71; 1.64 | 0.733 | 50.22 | 71.06 | 1.41 | 0.82; 2.43 | 0.207 |
| ***Sudan*** | 32.98 | 14.51 | 0.44 | 0.21; 0.90 | 0.021 | 5.65 | 3.21 | 0.57 | 0.22; 1.44 | 0.226 |
| ***Pooled estimate*** |  |  | **0.83** | **0.55; 1.26** | **0.388** |  |  | **0.84** | **0.52; 1.36** | **0.478** |
|  | **WAZ <-2** | | | | | | | | | |
|  | ***AR 6-23m*** | | ***RR boys vs. girls (ref)*** | | | ***AR 24-59m*** | | ***RR boys vs. girls (ref)*** | | |
|  | ***Girls*** | ***Boys*** | ***Β*** | ***95% CI*** | ***P*** | ***Girls*** | ***Boys*** | ***β*** | ***95% CI*** | ***P*** |
| ***DRC*** | 24.77 | 32.16 | 1.30 | 0.75; 2.26 | 0.353 | 11.52 | 11.19 | 0.97 | 0.54; 1.76 | 0.923 |
| ***Guinea Bissau*** | 71.09 | 53.49 | 0.75 | 0.37; 1.54 | 0.437 | 35.71 | 37.03 | 1.04 | 0.31; 3.46 | 0.953 |
| ***Indonesia*** | 40.58 | 43.07 | 1.06 | 0.65; 1.74 | 0.814 | 14.98 | 12.16 | 0.81 | 0.48; 1.37 | 0.435 |
| ***Nepal*** | 17.71 | 8.14 | 0.46 | 0.27; 0.79 | 0.004 | 3.8 | 5.85 | 1.54 | 0.82; 2.88 | 0.173 |
| ***Philippines*** | 23.43 | 23.02 | 0.98 | 0.72; 1.34 | 0.910 | 0 | 0 |  |  |  |
| ***Senegal*** | 55.39 | 55.55 | 1.0 | 0.71; 1.42 | 0.987 | 41.98 | 44.87 | 1.07 | 0.69; 1.64 | 0.762 |
| ***Sudan*** | 18.46 | 7.45 | 0.40 | 0.22; 0.75 | 0.003 | 1.86 | 1.99 | 1.07 | 0.57; 2.0 | 0.837 |
| ***Pooled estimate*** |  |  | **0.82** | **0.61; 1.09** | **0.176** |  |  | **1.05** | **0.82; 1.33** | **0.708** |
|  | **HAZ <-2** | | | | | | | | | |
|  | ***AR 6-23m*** | | ***RR boys vs. girls (ref)*** | | | ***AR 24-59m*** | | ***RR boys vs. girls (ref)*** | | |
|  | ***Girls*** | ***Boys*** | ***Β*** | ***95% CI*** | ***P*** | ***Girls*** | ***Boys*** | ***β*** | ***95% CI*** | ***P*** |
| ***DRC*** | 16.13 | 25.13 | 1.56 | 0.96; 2.53 | 0.072 | 8.03 | 6.43 | 0.80 | 0.48; 1.33 | 0.393 |
| ***Guinea Bissau*** | 65.65 | 51.59 | 0.79 | 0.45; 1.37 | 0.396 | 28.42 | 20.68 | 0.73 | 0.30; 1.74 | 0.473 |
| ***Indonesia*** | 27.58 | 31.48 | 1.14 | 0.73; 1.79 | 0.563 | 12.44 | 8.24 | 0.66 | 0.43; 1.03 | 0.066 |
| ***Nepal*** | 12.66 | 7.11 | 0.56 | 0.32; 0.98 | 0.038 | 3.8 | 4.39 | 1.06 | 0.67; 2.00 | 0.605 |
| ***Philippines*** | 16.61 | 15.1 | 0.91 | 0.67; 1.23 | 0.540 | 0 | 0 |  |  |  |
| ***Senegal*** | 66.13 | 56.93 | 0.86 | 0.52; 1.43 | 0.562 | 39.54 | 32.97 | 0.83 | 0.53; 1.30 | 0.423 |
| ***Sudan*** | 12.5 | 5.63 | 0.51 | 0.25; 0.83 | 0.008 | 1.4 | 1.37 | 0.97 | 0.52; 1.84 | 0.936 |
| ***Pooled estimate*** |  |  | **0.88** | **0.67; 1.14** | **0.318** |  |  | **0.82** | **0.66; 1.02** | **0.070** |

AR represents the absolute risk of death in the exposed group per 1000 children; RR represents the relative risk of death in boys vs. girls (ref); pooled estimate represents the weighted pooled estimates from the meta-analysis

**Supplementary Table 4: Absolute risk of mortality associated with severe anthropometric deficits by age and relative risk of mortality in younger compared to older children.**

|  | **MUAC <115mm** | | | | | | | | | | | | | | | |
| --- | --- | --- | --- | --- | --- | --- | --- | --- | --- | --- | --- | --- | --- | --- | --- | --- |
|  | ***Both sexes*** | | | | | ***Girls*** | | | | | | ***Boys*** | | | | |
|  | ***AR 6-23m*** | ***AR 24-59m*** | ***RR 6-23m vs. 24-59m (ref)*** | ***95% CI*** | ***P*** | ***AR 6-23m*** | ***AR 24-59m*** | ***RR 6-23m vs. 24-59m (ref)*** | ***95% CI*** | ***P*** | ***AR 6-23m*** | | ***AR 24-59m*** | ***RR 6-23m vs. 24-59m (ref)*** | ***95% CI*** | ***P*** |
| ***DRC*** | 34.30 | 33.07 | 1.04 | 0.58; 1.85 | 0.902 | 29.47 | 37.31 | 0.79 | 0.36; 1.72 | 0.551 | 41.03 | | 28.46 | 1.44 | 0.60; 3.45 | 0.408 |
| ***Nepal*** | 29.67 | 70.0 | 0.42 | 0.18; 0.98 | 0.040 | 36.72 | 30.77 | 1.19 | 0.28; 5.05 | 0.810 | 14.22 | | 142.86 | 0.10 | 0.02; 0.40 | <0.001 |
| ***Senegal*** | 112.30 | 133.3 | 0.84 | 0.42; 1.70 | 0.634 | 129.41 | 108.70 | 1.19 | 0.44; 3.22 | 0.730 | 98.04 | | 172.41 | 0.57 | 0.21; 1.53 | 0.267 |
| ***Pooled estimate*** |  |  | **0.77** | **0.47; 1.26** | **0.302** |  |  | **0.96** | **0.55; 1.69** | **0.888** |  | |  | **0.49** | **0.13; 1.90** | **0.301** |
|  | **WHZ <-3** | | | | | | | | | | | | | | | |
|  | ***Both sexes*** | | | | | ***Girls*** | | | | | | ***Boys*** | | | | |
|  | ***AR 6-23m*** | ***AR 24-59m*** | ***RR 6-23m vs. 24-59m (ref)*** | ***95% CI*** | ***P*** | ***AR 6-23m*** | ***AR 24-59m*** | ***RR 6-23m vs. 24-59m (ref)*** | ***95% CI*** | ***P*** | ***AR 6-23m*** | | ***AR 24-59m*** | ***RR 6-23m vs. 24-59m (ref)*** | ***95% CI*** | ***P*** |
| ***DRC*** | 95.89 | 66.67 | 1.44 | 0.39; 5.28 | 0.580 | 35.71 | 100.0 | 0.36 | 0.03; 3.67 | 0.364 | 133.33 | | 40.0 | 3.33 | 0.42; 26.15 | 0.212 |
| ***Guinea Bissau*** | 193.55 | 83.33 | 2.32 | 0.31; 17.32 | 0.380 | 166.67 | 0 |  |  |  | 210.53 | | 166.67 | 1.26 | 0.17; 9.24 | 0.815 |
| ***Indonesia*** | 50.0 | 98.04 | 0.51 | 0.14; 1.81 | 0.289 | 38.46 | 200.0 | 0.19 | 0.02; 1.59 | 0.081 | 55.56 | | 32.26 | 1.72 | 0.19; 15.85 | 0.625 |
| ***Nepal*** | 25.46 | 37.88 | 0.67 | 0.24; 1.90 | 0.452 | 39.80 | 18.52 | 2.15 | 0.27; 16.81 | 0.452 | 12.99 | | 51.28 | 0.25 | 0.06; 1.11 | 0.049 |
| ***Philippines*** | 110.31 | 0 |  |  |  | 104.17 | 0 |  |  |  | 113.55 | | 0 |  |  |  |
| ***Senegal*** | 95.79 | 102.74 | 0.93 | 0.51; 1.71 | 0.821 | 126.21 | 115.38 | 1.09 | 0.49; 2.43 | 0.825 | 75.95 | | 88.24 | 0.86 | 0.34; 2.20 | 0.754 |
| ***Sudan*** | 50.28 | 16.11 | 3.12 | 1.49; 6.54 | 0.002 | 77.52 | 20.33 | 3.81 | 1.33; 10.92 | 0.007 | 34.93 | | 13.73 | 2.54 | 0.89; 7.24 | 0.070 |
| ***Pooled estimate*** |  |  | **1.21** | **0.66; 2.22** | **0.540** |  |  | **1.19** | **0.45; 3.13** | **0.728** |  | |  | **1.17** | **0.55; 2.48** | **0.690** |
|  | **WAZ <-3** | | | | | | | | | | | | | | | |
|  | ***Both sexes*** | | | | | ***Girls*** | | | | | | ***Boys*** | | | | |
|  | ***AR 6-23m*** | ***AR 24-59m*** | ***RR 6-23m vs. 24-59m (ref)*** | ***95% CI*** | ***P*** | ***AR 6-23m*** | ***AR 24-59m*** | ***RR 6-23m vs. 24-59m (ref)*** | ***95% CI*** | ***P*** | ***AR 6-23m*** | | ***AR 24-59m*** | ***RR 6-23m vs. 24-59m (ref)*** | ***95% CI*** | ***P*** |
| ***DRC*** | 41.67 | 21.76 | 1.91 | 1.08; 3.40 | 0.025 | 35.35 | 25.21 | 1.40 | 0.56; 3.51 | 0.469 | 45.45 | | 18.93 | 2.40 | 1.12; 5.17 | 0.021 |
| ***Guinea Bissau*** | 86.96 | 66.67 | 1.30 | 0.48; 3.56 | 0.602 | 87.72 | 45.45 | 1.93 | 0.39; 9.48 | 0.407 | 86.42 | | 96.77 | 0.89 | 0.25; 3.24 | 0.864 |
| ***Indonesia*** | 48.31 | 27.33 | 1.77 | 1.01; 3.09 | 0.043 | 40.70 | 25.24 | 1.61 | 0.65; 3.98 | 0.297 | 53.72 | | 29.30 | 1.83 | 0.90; 3.75 | 0.093 |
| ***Nepal*** | 20.53 | 7.93 | 2.59 | 1.53; 4.38 | <0.001 | 30.40 | 4.90 | 6.20 | 2.73; 14.10 | <0.001 | 11.07 | | 11.48 | 0.96 | 0.44; 2.11 | 0.928 |
| ***Philippines*** | 49.55 | 0 |  |  |  | 46.40 | 0 |  |  |  | 51.98 | | 0 |  |  |  |
| ***Senegal*** | 74.49 | 78.51 | 0.95 | 0.64; 1.40 | 0.793 | 79.24 | 72.58 | 1.09 | 0.62; 1.92 | 0.761 | 70.42 | | 84.76 | 0.83 | 0.48; 1.43 | 0.504 |
| ***Sudan*** | 24.62 | 3.85 | 6.40 | 3.80; 10.76 | <0.001 | 33.39 | 4.34 | 7.69 | 3.88; 15.24 | <0.001 | 17.78 | | 3.36 | 5.29 | 2.38; 11.77 | <0.001 |
| ***Pooled estimate*** |  |  | **2.05** | **1.13; 3.73** | **0.018** |  |  | **2.52** | **1.16; 5.47** | **0.020** |  | |  | **1.62** | **0.89; 2.92** | **0.113** |
|  | **HAZ <-3** | | | | | | | | | | | | | | | |
|  | ***Both sexes*** | | | | | ***Girls*** | | | | | | ***Boys*** | | | | |
|  | ***AR 6-23m*** | ***AR 24-59m*** | ***RR 6-23m vs. 24-59m (ref)*** | ***95% CI*** | ***P*** | ***AR 6-23m*** | ***AR 24-59m*** | ***RR 6-23m vs. 24-59m (ref)*** | ***95% CI*** | ***P*** | ***AR 6-23m*** | | ***AR 24-59m*** | ***RR 6-23m vs. 24-59m (ref)*** | ***95% CI*** | ***P*** |
| ***DRC*** | 26.48 | 10.93 | 2.42 | 1.58; 3.72 | <0.001 | 17.21 | 11.44 | 1.50 | 0.69; 3.28 | 0.304 | 31.80 | | 10.54 | 3.02 | 1.77; 5.15 | <0.001 |
| ***Guinea Bissau*** | 77.78 | 34.19 | 2.28 | 1.03; 5.04 | 0.036 | 98.36 | 31.01 | 3.17 | 1.05; 9.57 | 0.029 | 60.81 | | 38.10 | 1.60 | 0.50; 5.05 | 0.420 |
| ***Indonesia*** | 47.66 | 13.11 | 3.64 | 2.48; 5.33 | <0.001 | 43.88 | 16.20 | 2.71 | 1.54; 4.77 | <0.001 | 50.32 | | 10.40 | 4.84 | 2.82; 8.29 | <0.001 |
| ***Nepal*** | 12.92 | 5.70 | 2.27 | 1.44; 3.58 | <0.001 | 19.82 | 4.52 | 4.38 | 2.31; 8.33 | <0.001 | 7.35 | | 6.86 | 1.07 | 0.52; 2.19 | 0.850 |
| ***Philippines*** | 20.84 | 0 |  |  |  | 22.24 | 0 |  |  |  | 19.90 | | 0 |  |  |  |
| ***Senegal*** | 68.09 | 57.02 | 1.19 | 0.68; 2.09 | 0.535 | 102.56 | 57.88 | 1.77 | 0.80; 3.92 | 0.158 | 50.96 | | 56.37 | 0.90 | 0.41; 1.98 | 0.800 |
| ***Sudan*** | 13.59 | 1.88 | 7.22 | 4.29; 12.16 | <0.001 | 21.61 | 2.13 | 10.16 | 5.22; 19.79 | <0.001 | 7.48 | | 1.64 | 4.55 | 1.94; 10.70 | <0.001 |
| ***Pooled estimate*** |  |  | **2.74** | **1.74; 4.32** | **<0.001** |  |  | **3.23** | **1.82; 5.73** | **<0.001** |  | |  | **2.24** | **1.23; 4.06** | **0.008** |

AR represents the absolute risk of death in the exposed group per 1000 children; RR represents the relative risk of death in young (6-23 months) vs. older (24-59 months; ref) age; pooled estimate represents the weighted pooled estimates from the meta-analysis

**Supplementary table 4a: Absolute risk of mortality associated with severe anthropometric deficits by sex and relative risk of mortality in younger compared to older children.**

|  | **MUAC <115mm** | | | | | | | | | |
| --- | --- | --- | --- | --- | --- | --- | --- | --- | --- | --- |
|  | ***AR 6-23m*** | | ***RR boys vs. girls (ref)*** | | | ***AR 24-59m*** | | ***RR boys vs. girls (ref)*** | | |
|  | ***Girls*** | ***Boys*** | ***β*** | ***95% CI*** | ***P*** | ***Girls*** | ***Boys*** | ***β*** | ***95% CI*** | ***P*** |
| ***DRC*** | 29.47 | 41.03 | 1.39 | 0.70; 2.75 | 0.339 | 37.31 | 28.46 | 0.76 | 0.29; 1.97 | 0.575 |
| ***Nepal*** | 36.72 | 14.22 | 0.39 | 0.11; 1.31 | 0.110 | 30.77 | 142.86 | 4.64 | 0.95; 22.7 | 0.036 |
| ***Senegal*** | 129.41 | 98.04 | 0.76 | 0.34; 1.70 | 0.499 | 108.70 | 172.41 | 1.59 | 0.50; 5.00 | 0.429 |
| ***Pooled estimate*** |  |  | **0.86** | **0.44; 1.66** | **0.644** |  |  | **1.51** | **0.58; 3.93** | **0.394** |
|  | **WHZ <-3** | | | | | | | | | |
|  | ***AR 6-23m*** | | ***RR boys vs. girls (ref)*** | | | ***AR 24-59m*** | | ***RR boys vs. girls (ref)*** | | |
|  | ***Girls*** | ***Boys*** | ***β*** | ***95% CI*** | ***P*** | ***Girls*** | ***Boys*** | ***β*** | ***95% CI*** | ***P*** |
| ***DRC*** | 35.71 | 133.33 | 3.73 | 0.47; 29.40 | 0.168 | 100.0 | 40.0 | 0.40 | 0.04; 4.10 | 0.423 |
| ***Guinea Bissau*** | 166.67 | 210.53 | 1.26 | 0.27; 5.87 | 0.763 | 0 | 166.67 |  |  |  |
| ***Indonesia*** | 38.46 | 55.56 | 1.44 | 0.16; 13.22 | 0.743 | 200.0 | 32.26 | 0.16 | 0.02; 1.34 | 0.049 |
| ***Nepal*** | 39.80 | 12.99 | 0.33 | 0.09; 1.21 | 0.078 | 18.52 | 51.28 | 2.77 | 0.32; 24.10 | 0.332 |
| ***Philippines*** | 104.17 | 113.55 | 1.09 | 0.61; 1.95 | 0.771 | 0 | 0 |  |  |  |
| ***Senegal*** | 126.21 | 75.95 | 0.60 | 0.29; 1.27 | 0.177 | 115.38 | 88.24 | 0.76 | 0.29; 2.04 | 0.590 |
| ***Sudan*** | 77.52 | 34.93 | 0.45 | 0.18; 1.11 | 0.077 | 20.33 | 13.73 | 0.68 | 0.21; 2.19 | 0.511 |
| ***Pooled estimate*** |  |  | **0.77** | **0.49; 1.21** | **0.257** |  |  | **0.68** | **0.36; 1.29** | **0.237** |
|  | **WAZ <-3** | | | | | | | | | |
|  | ***AR 6-23m*** | | ***RR boys vs. girls (ref)*** | | | ***AR 24-59m*** | | ***RR boys vs. girls (ref)*** | | |
|  | ***Girls*** | ***Boys*** | ***β*** | ***95% CI*** | ***P*** | ***Girls*** | ***Boys*** | ***β*** | ***95% CI*** | ***P*** |
| ***DRC*** | 35.35 | 45.45 | 1.29 | 0.53; 3.10 | 0.574 | 25.21 | 18.93 | 0.75 | 0.33; 1.69 | 0.487 |
| ***Guinea Bissau*** | 87.72 | 86.42 | 0.99 | 0.33; 2.95 | 0.979 | 45.45 | 96.77 | 2.13 | 0.38; 12.0 | 0.380 |
| ***Indonesia*** | 40.70 | 53.72 | 1.32 | 0.54; 3.24 | 0.543 | 25.24 | 29.30 | 1.16 | 0.56; 2.39 | 0.685 |
| ***Nepal*** | 30.40 | 11.07 | 0.36 | 0.18; 0.72 | 0.003 | 4.90 | 11.48 | 2.34 | 0.95; 5.78 | 0.057 |
| ***Philippines*** | 46.40 | 51.98 | 1.12 | 0.75; 1.68 | 0.580 | 0 | 0 |  |  |  |
| ***Senegal*** | 79.24 | 70.42 | 0.89 | 0.54; 1.45 | 0.638 | 72.58 | 84.76 | 1.17 | 0.63; 2.15 | 0.619 |
| ***Sudan*** | 33.39 | 17.78 | 0.53 | 0.27; 1.07 | 0.072 | 4.34 | 3.36 | 0.77 | 0.35; 1.70 | 0.521 |
| ***Pooled estimate*** |  |  | **0.84** | **0.59; 1.19** | **0.316** |  |  | **1.13** | **0.81; 1.57** | **0.460** |
|  | **HAZ <-3** | | | | | | | | | |
|  | ***AR 6-23m*** | | ***RR boys vs. girls (ref)*** | | | ***AR 24-59m*** | | ***RR boys vs. girls (ref)*** | | |
|  | ***Girls*** | ***Boys*** | ***β*** | ***95% CI*** | ***P*** | ***Girls*** | ***Boys*** | ***β*** | ***95% CI*** | ***P*** |
| ***DRC*** | 17.21 | 31.80 | 1.85 | 0.88; 3.87 | 0.098 | 11.44 | 10.54 | 0.92 | 0.51; 1.66 | 0.787 |
| ***Guinea Bissau*** | 98.36 | 60.81 | 0.62 | 0.27; 1.42 | 0.252 | 31.01 | 38.10 | 1.23 | 0.31; 4.80 | 0.767 |
| ***Indonesia*** | 43.88 | 50.32 | 1.15 | 0.66; 2.00 | 0.630 | 16.20 | 10.40 | 0.64 | 0.37; 1.11 | 0.109 |
| ***Nepal*** | 19.82 | 7.35 | 0.37 | 0.18; 0.75 | 0.004 | 4.52 | 6.86 | 1.52 | 0.79; 2.90 | 0.205 |
| ***Philippines*** | 22.24 | 19.90 | 0.89 | 0.58; 1.37 | 0.611 | 0 | 0 |  |  |  |
| ***Senegal*** | 102.56 | 50.96 | 0.50 | 0.19; 1.27 | 0.139 | 57.88 | 56.37 | 0.97 | 0.54; 1.77 | 0.931 |
| ***Sudan*** | 21.61 | 7.48 | 0.35 | 0.16; 0.73 | 0.003 | 2.13 | 1.64 | 0.77 | 0.35; 1.70 | 0.519 |
| ***Pooled estimate*** |  |  | **0.72** | **0.47; 1.11** | **0.134** |  |  | **0.92** | **0.70; 1.21** | **0.550** |

AR represents the absolute risk of death in the exposed group per 1000 children; RR represents the relative risk of death in boys vs. girls (ref); pooled estimate represents the weighted pooled estimates from the meta-analysis
